# Cadmium Induces the Expression of Grp78, an Endoplasmic Reticulum Molecular Chaperone, in LLC-PK1 Renal Epithelial Cells

**DOI:** 10.1289/ehp.8920

**Published:** 2006-02-16

**Authors:** Fang Liu, Kiyoshi Inageda, Gen Nishitai, Masato Matsuoka

**Affiliations:** Department of Hygiene and Public Health I, School of Medicine, Tokyo Women’s Medical University, Tokyo, Japan

**Keywords:** ATF4, cadmium, eIF2α, endoplasmic reticulum stress, Grp78, heavy metal, LLC-PK1 cells, siRNA

## Abstract

To reveal the effects of cadmium exposure on the endoplasmic reticulum (ER) stress response, we examined the expression and function of 78-kDa glucose-regulated protein (Grp78), an ER-resident molecular chaperone, in LLC-PK1 cells. In cells treated with 10 μM cadmium chloride, Grp78 protein levels increased after 6 hr and remained elevated at 24 hr. When cells were incubated with 1–20 μM CdCl_2_ for 6 hr, Grp78 increased in a dose-dependent manner. In addition, Grp78 mRNA levels were elevated in response to CdCl_2_ exposure. After exposure to 10 μM CdCl_2_, the levels of activating transcription factor 4 (ATF4) were increased at 2 hr, with a further enhancement after that; this accumulation followed the transient but marked phosphorylation of the α subunit of eukaryotic translation initiation factor 2 (eIF2α) on serine 51. Although ATF4 mRNA levels increased mildly by CdCl_2_ exposure, treatment with actinomycin D did not suppress CdCl_2_-induced accumulation of ATF4 protein, suggesting the involvement of posttranscriptional and, in part, transcriptional mechanisms. Compared with other heavy-metal compounds such as manganese chloride, zinc chloride, mercuric chloride, and lead chloride, CdCl_2_ could increase the levels of Grp78, ATF4, and the phosphorylated form of eIF2α more markedly without definite cellular damage. The silencing of *Grp78* expression using short-interference RNA enhanced CdCl_2_-induced cellular damage. These results show that cadmium induces the expression of *Grp78* probably via phosphorylation of eIF2α and resultant translation of ATF4, and this ER stress response plays a role in protection against cadmium cytotoxicity in this renal epithelial cell.

Cadmium is an important occupational and environmental pollutant that causes damage to various organs, especially renal proximal tubular cells (Goering et al. 1995). Cadmium has been reported to induce apoptotic cell death in proximal tubules of experimental animals (Hamada et al. 1997). However, the molecular mechanisms responsible for cadmium-induced damage and subsequent regeneration of tubular epithelium have not been fully clarified. It is important to determine the subcellular compartments that respond to cellular stress induced by cadmium exposure and affect diverse areas of cellular function such as signal transduction, gene expression, cell survival, and death.

The endoplasmic reticulum (ER), an essential intracellular organelle, is responsible for the synthesis, posttranslational modification, and delivery of biologically active proteins to their proper target sites within the cell and the extracellular milieu, as well as for Ca^2+^ homeostasis (Brostrom and Brostrom 2003; Schröder and Kaufman 2005). The accumulation of unfolded proteins in the lumen of the ER causes ER stress and induces a coordinated adaptive program called the unfolded protein response (UPR). The UPR alleviates stress by up-regulating protein folding and degradation pathways in the ER and by inhibiting protein synthesis (Mori 2000; Rutkowski and Kaufman 2004). The UPR induces the expression of 78-kDa glucose-regulated protein [Grp78; also known as immunoglobulin heavy-chain–binding protein (BiP)], an ER-resident molecular chaperone that prevents the aggregation of unfolded or misfolded proteins so that they can be properly refolded (Brostrom and Brostrom 2003; Lee 2001; Schröder and Kaufman 2005). Grp78 is also the member of heat shock protein 70 (HSP70) family of cytoplasmic chaperones (Brostrom and Brostrom 2003). The *Grp78* gene is induced by the various perturbations of ER function such as glucose starvation, expression of misfolded or underglycosylated proteins, treatment with reducing agents, and depletion of Ca^2+^ stores in the ER (Lee 2001).

With respect to heavy metals, cadmium has been reported to induce the expression of Grp78 in mIMCD3 murine inner medullary collecting duct cells (Santos et al. 1998; Zhang et al. 1999), NIH3T3 mouse fibroblasts (Sugisawa et al. 2004), A549 human pulmonary epithelial type II cells (Croute et al. 2005), RLE rat lung epithelial cells (Timblin et al. 1998), and HeLa cells (Cigliano et al. 1996), but not in HepG2 human hepatoma cells (Mumtaz et al. 2002; Tchounwou et al. 2001; Tully et al. 2000) or platyfish culture cells (Yamashita et al. 2004). These findings suggest that the expression of Grp78 depends on the type of cell exposed to cadmium. However, alterations of Grp78 expression in the proximal tubular cells, which are one of the major targets damaged by cadmium exposure, have not been studied. Furthermore, neither the mechanism nor the biologic significance of cadmium-induced Grp78 expression is known. We therefore examined whether treatment with cadmium chloride and other heavy-metal compounds can induce the expression of Grp78 in LLC-PK1 cells, an established porcine renal epithelial cell line with characteristics of the proximal tubule (Gstraunthaler 1988). Because activating transcription factor 4 (ATF4) and ATF6 have been reported to be responsible for transcription of the *Grp78* gene (Luo et al. 2003; Roybal et al. 2004; Rutkowski and Kaufman 2004), we determined the expression of ATF4 and the phosphorylation of eukaryotic translation initiation factor 2 on serine 51 of its α subunit (eIF2α), an upstream regulator of ATF4 expression (Harding et al. 2000). Using short-interference RNA (siRNA) against the porcine *Grp78* gene, effects of Grp78 knockdown on the cytotoxicity of CdCl_2_ were also examined in this renal epithelial cell.

## Materials and Methods

### Cell culture

LLC-PK1 cells were obtained from Health Science Research Resources Bank (Japan Health Sciences Foundation, Osaka, Japan) and grown in medium 199 supplemented with 3% heat-inactivated fetal bovine serum, 100 units/mL penicillin, and 100 μg/mL streptomycin (GIBCO; Invitrogen Corp., Carlsband, CA, USA) in a humidified atmosphere of 5% CO_2_, 95% air at 37°C. For each experiment, exponentially growing LLC-PK1 cells were plated at 5 × 10^5^ or 2.5 × 10^5^ cells/well in 6-well culture plates, 6 × 10^4^ cells/well in 24-well culture plates, or 1 × 10^4^ cells/well in 96-well culture plates, and cultured for 1 day before the experiments.

### siRNA transfection

Duplexed stealth siRNA targeted against the porcine *Grp78* gene (GenBank accession no. X92446; GenBank, National Center for Biotechnology Information, Bethesda, MD, USA) was synthesized by Invitrogen. The sequence of 25-mer siRNA was 5′-GGGAAAGAAGGU-UACUCAUGCAGUU-3′. siRNA was transfected into LLC-PK1 cells grown in 6-, 24-, or 96-well culture plates (50% confluence) using Lipofectamine 2000 (Invitrogen) following the manufacturer’s instructions. After incubating for 12 hr, cells were washed with medium 199 and used for the experiments. Transfection efficiency was evaluated using a fluorescent oligonucleotide and estimated to be > 80%.

### Treatment with metals

CdCl_2_, zinc chloride, mercuric chloride, and lead chloride were obtained from Wako Pure Chemical Industries, Ltd. (Osaka, Japan), and manganese chloride was from Sigma Chemical Co. (St. Louis, MO, USA). The stock solutions were prepared by dissolving each metal compound in water and sterilizing the solution by filtration. LLC-PK1 cells (90% confluence) were incubated with serum-free medium containing the appropriate concentration of CdCl_2_ or other metals for 6 hr at 37°C. In the time-course study, cells were incubated with 10 μM CdCl_2_ for 1–24 hr. Untreated control cells were incubated with serum-free medium alone and treated identically to the cells exposed to metals. Actinomycin D, cycloheximide, and thapsigargin (Sigma) were dissolved in dimethyl sulfoxide (DMSO). LLC-PK1 cells were incubated with serum-free medium containing each chemical or DMSO at the same concentration used (0.1, 0.05, or 0.03%).

### Western immunoblotting

At the end of the incubation, cells were washed with phosphate-buffered saline and lysed with sodium dodecyl sulfate (SDS)–polyacrylamide gel Laemmli sample buffer. Cell lysates were collected, sonicated, and boiled for 5 min. Protein concentration was determined with the RC DC Protein Assay (Bio-Rad Laboratories, Inc., Hercules, CA, USA). Equal amounts of protein (10 or 20 μg) were subjected to SDS-PAGE on a 10% polyacrylamide gel and transferred to a nitrocellulose membrane (Hybond-ECL; Amersham Pharmacia Biotech, Little Chalfont, Buckinghamshire, UK). The membrane was blocked with 5% nonfat milk or bovine serum albumin in Tris-buffered saline containing 0.1% Tween 20 for 1 hr at room temperature. The antibodies used were Grp78 (76-E6), cAMP-responsive element (CRE) binding protein 2 CREB2 (also known as ATF4; C-20), and actin (I-19) (all from Santa Cruz Biotechnology, Inc., Santa Cruz, CA, USA); 94-kDa glucose-regulated protein (Grp94) and HSP70 (both from Stressgen Bioreagents, Victoria, British Columbia, Canada); and phosphorylated eIF2α (phospho-eIF2α; Ser51) and total eIF2α (both from Cell Signaling Technology, Inc., Beverly, MA, USA). The membrane was incubated overnight at 4°C with the primary antibody, and protein was detected with a Phototope-HRP Western blot detection kit (Cell Signaling Technology) or a SuperSignal West Femto Maximum Sensitivity Substrate kit (Pierce Chemical Co., Rockford, IL, USA). After immunodetection, some blots were incubated with Restore Western Blot Stripping Buffer (Pierce) for 30 min at room temperature and reprobed with actin antibody. The bands on the developed film were quantified with NIH Image (version 1.62; National Institutes of Health, Bethesda, MD, USA). The density of each band was normalized to that of actin.

### RT-PCR

The reverse-transcription polymerase chain reaction (RT-PCR) analysis for the semiquantification of mRNA was carried out as described previously (Matsuoka and Igisu 1996). Total RNA was isolated using Trizol reagent (Invitrogen), and 0.5 μg of total RNA from each sample was used for cDNA synthesis using the first-strand cDNA synthesis kit (Roche Applied Science, Penzberg, Germany). Equal volumes (1 μL) of the resulting cDNA served as templates for subsequent PCR reactions using DNA polymerase KOD Dash (Toyobo, Osaka, Japan). The primers for *Grp78* were designed from the porcine Grp78 mRNA sequence to yield an expected product of 656 bp. The sense primer sequence was 5′-GCACCACCTAC TCGTGCGTT-3′ (bases 245–264), and the antisense primer was 5′-ACCCAGGTGAGT ATCTCCGTTAG-3′ (bases 878–900). The sequences of *ATF4* primers (Kato et al. 1999) were 5′-CCAGGTTGCCCCCTTTACGTTCTTG-3′ (sense) and 5′-GTTCTGCTCCATCTTCTTCAGCTTC-3′ (antisense), which yielded a 412-bp product corresponding to nucleotides 678–1,089 on the porcine *ATF4* gene. The sequences of β-actin primers (Yano et al. 2004) were 5′-TGAGACCTT CAACACGCCG-3′ (sense) and 5′-ATG GTGATGACCTGCCCGTC-3′ (antisense), which yielded a 378-bp product corresponding to nucleotides 6–383 on the porcine β-actin gene. An aliquot of PCR products (10 μL) was run on a 2% agarose gel containing 0.5 μg/mL ethidium bromide. The densities of each band were recorded with an Image Saver HR (AE-6905H; Atto, Tokyo, Japan) and quantified using NIH Image software. For mRNA analysis, density of each product was normalized to that of β-actin.

### Trypan blue exclusion assay

Culture medium was aspirated and reserved. After trypsinization, cells were suspended in medium 199, and the culture medium was returned. The mixture was centrifuged to concentrate the cells. Cellular suspension and 0.4% trypan blue in Hank’s balanced salt solution were mixed, and the number of viable cells was counted using a hemacytometer. The percentage of viable cells (cell viability) was calculated as 100 × [(unstained cells)/(stained + unstained cells)].

### LDH assay

The activity of lactate dehydrogenase (LDH) in the supernatant of cells was determined using a cytotoxicity detection kit (LDH; Roche) according to the manufacturer’s instructions. The results were expressed as the percentage of the maximum amount of LDH released from samples that had been treated with 1% Triton X-100 (percentage release).

### Statistical analysis

Results were expressed as the mean ± SD. Statistical significance was determined by one-way analysis of variance followed by the Dunnett multiple comparison test. When two groups were compared, Student’s *t*-test or Welch’s *t*-test was used; *p* < 0.05 was considered statistically significant.

## Results

### Accumulation of Grp78 by CdCl_2_

**Figure 1:**
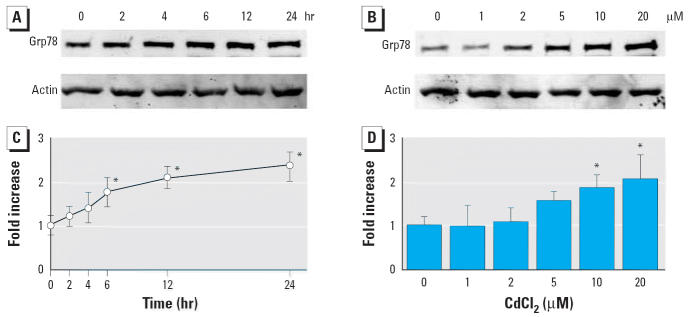
Time course (*A,C*) and dose effects (*B,D*) of CdCl_2_-induced accumulation of Grp78 protein in LLC-PK1 cells incubated with 10 μM CdCl_2_ for 2–24 hr (*A,C*) or with 0, 1, 2, 5, 10, or 20 μM CdCl_2_ for 6 hr (*B,D*). (*A,B*) Cell lysates subjected to Western immunoblotting using Grp78 and actin antibodies. (*C,D*) Densitometric analyses of (*A* and *B*, respectively). Results shown are from representative analyses. Control values (0 hr or 0 μM) were set to 1; values are mean ± SD of four experiments. *p < 0.05 compared with control.

In LLC-PK1 cells treated with 10 μM CdCl_2_, the level of Grp78 increased significantly after 6 hr and remained elevated at 24 hr, whereas the actin level was not changed after 2- to 24-hr exposures (Figure 1A). When cells were incubated with 1–20 μM CdCl_2_ for 6 hr, the Grp78 level increased in a dose-dependent manner (Figure 1B). In contrast, the level of Grp94, which is also abundant in the ER lumen (Lee 2001), was not changed clearly in response to CdCl_2_ exposure (data not shown). After incubation with CdCl_2_ for 6 hr, the cell viability assayed with trypan blue exclusion was not changed at concentrations < 10 μM and was reduced by 36% at 20 μM CdCl_2_. Hereafter, cells were exposed to CdCl_2_ at a concentration of 10 μM.

### Induction of Grp78 gene expression by CdCl_2_

**Figure 2:**
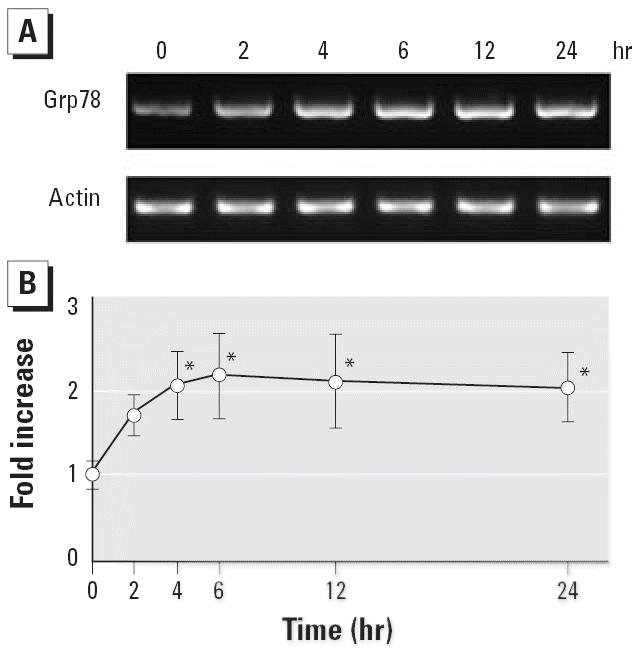
Induction of *Grp78* gene expression in LLC-PK1 cells incubated with 10 μM CdCl_2_ for 2–24 hr; total RNA was isolated and subjected to RT-PCR analysis using *Grp78* and β-actin primers. (*A*) Agarose gel stained with ethidium bromide. (*B*) *Grp78* gene expression shown by densitometric analysis of the gel in (*A*). The control value (0 hr) was set to 1. Values are mean ± SD of three experiments. *p < 0.05 compared with control.

PCR amplification with *Grp78* primers showed single bands of the predicted size (656 bp) on an agarose gel stained with ethidium bromide (Figure 2). Consistent with the increase of Grp78, Grp78 mRNA in LLC-PK1 cells treated with 10 μM CdCl_2_ began to increase significantly after 4 hr and peaked at 6 hr (Figure 2). The expression of β-actin did not change after treatment with CdCl_2_.

### Accumulation of ATF4 and phospho-eIF2α by CdCl_2_

**Figure 3:**
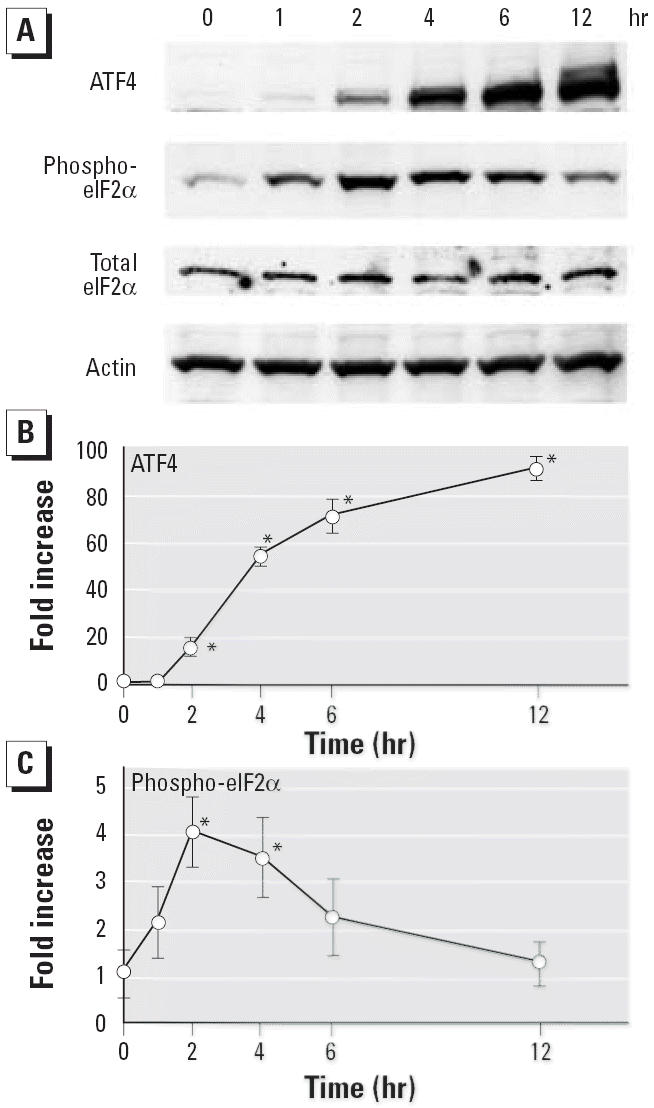
Accumulation of ATF4 and phospho-eIF2α by CdCl_2_ in LLC-PK1 cells incubated with 10 μM CdCl_2_ for 1–12 hr; cell lysates were subjected to Western immunoblotting (*A*) using ATF4, phospho-eIF2α, total eIF2α, and actin antibodies. Densitometric analysis of the Western blot in (*A*) showing ATF4 (*B*) and phospho-eIF2α (*C*). Results shown are representative analyses. The control value (0 hr) was set to 1. Values are mean ± SD of three experiments. *p < 0.01 compared with control.

ATF4 levels increased clearly after 2 hr, and this elevation became more marked as incubation time increased (Figure 3). In contrast to ATF4 levels, the level of ATF6 detected at 90 kDa was not changed, and its 50-kDa fragment did not appear after 2–24 hr of exposure (data not shown). Phospho-eIF2α levels began to increase after 1 hr, peaked at 2 hr, and then returned to the control level at 12 hr (Figure 3). In contrast, the endogenous level of total eIF2α did not change through the incubation periods examined. Thus, treatment of LLC-PK1 cells with CdCl_2_ induces transient phosphorylation of eIF2α first, followed by progressive accumulation of ATF4 as time of exposure increases.

### Posttranscriptional regulation of ATF4 expression

**Figure 4:**
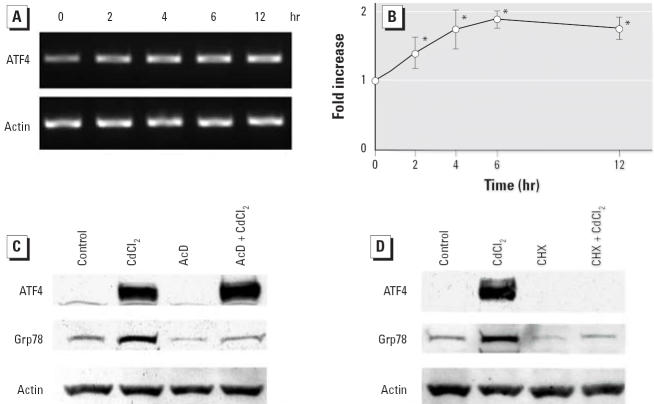
Posttranscriptional regulation of ATF4 expression. (*A*) LLC-PK1 cells were incubated with 10 μM CdCl_2_ for 2–12 hr, and total RNA was isolated and subjected to RT-PCR analysis using *ATF4* and β-actin primers. (*B*) Densitometric analysis of (*A*). The control value (0 hr) was set to 1; values are mean ± SD of three experiments. (*C*,*D*) LLC-PK1 cells were incubated for 6 hr with (*C*) 10 μM CdCl_2_, 1 μg/mL actinomycin D (AcD), or 1 μg/mL AcD plus 10 μM CdCl_2_, or (*D*) 10 μM CdCl_2_, 10 μg/mL cycloheximide (CHX), or 10 μg/mL CHX plus 10μM CdCl_2_. The untreated cells (control) were incubated with serum-free medium containing DMSO at the concentration used in the treated cells (0.05 or 0.1%). Cell lysates were subjected to Western immunoblotting using ATF4, Grp78, and actin antibodies. Results shown are representative of three experiments. *p < 0.01 compared with control.

After exposure to CdCl_2_, the level of ATF4 mRNA increased after 2 hr, whereas β-actin mRNA levels were not changed after 2–12 hr exposures (Figure 4A). However, the increase of ATF4 mRNA level was < 2-fold and peaked at 6 hr (Figure 4B), suggesting that posttranscriptional mechanisms regulate ATF4 expression in LLC-PK1 cells treated with CdCl_2_. Therefore, effects of actinomycin D and cycloheximide on CdCl_2_-induced expression of ATF4 and Grp78 were examined. Treatment of actinomycin D, an inhibitor of transcription, suppressed the accumulation of Grp78 but not of ATF4 (Figure 4C). On the other hand, treatment with cycloheximide, a protein synthesis inhibitor, abolished the expression of both proteins in cells exposed to 10μM CdCl_2_ for 6 hr (Figure 4D).

### Effects of heavy metals on the expression of Grp78, ATF4, and phospho-eIF2α proteins

**Figure 5:**
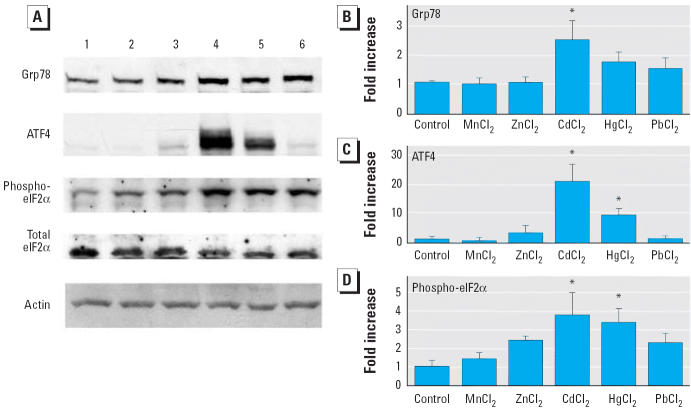
Expression of Grp78, ATF4, and phospho-eIF2α proteins in LLC-PK1 cells incubated with 10 μM of each heavy-metal compound for 6 hr. (*A*) Representative immunoblot obtained with Grp78, ATF4, phospho-eIF2α, total eIF2α, and actin antibodies. Lane 1, control; lane 2, MnCl_2_; lane 3, ZnCl_2_; lane 4, CdCl_2_; lane 5, HgCl_2_; lane 6, PbCl_2_. (*B–D*) Densitometric analysis showing (*B*) Grp78, (*C*) ATF4, and (*D*) phospho-eIF2α. The control value (without metals) was set to 1; values are mean + SD of three (for ATF4 and phospho-eIF2α) or four (for Grp78) experiments. *p < 0.05 compared with control.

Among the heavy metals examined, only CdCl_2_ treatment increased the level of Grp78 protein significantly in LLC-PK1 cells (Figure 5A,B). The levels of ATF4 and phospho-eIF2α proteins were elevated in cells exposed to CdCl_2_ and HgCl_2_, both of which are nephrotoxic heavy-metal compounds. The increase of ATF4 level was more marked in cells treated with CdCl_2_ than with HgCl_2_ (*p* < 0.05). However, no significant increases were found in cells treated with MnCl_2_, ZnCl_2_, or PbCl_2_ at the same concentration (10 μM) for 6 hr (Figure 5A,B). The cell viability assayed with trypan blue exclusion was 98.7 ± 0.7% for MnCl_2_, 98.3 ± 0.3% for ZnCl_2_, 95.6 ± 1.7% for CdCl_2_, 55.6 ± 7.8% for HgCl_2_, and 97.3 ± 0.7% for PbCl_2_ (mean ± SD of three experiments).

### Effects of Grp78 knockdown on the cytotoxicity of CdCl_2_

**Figure 6:**
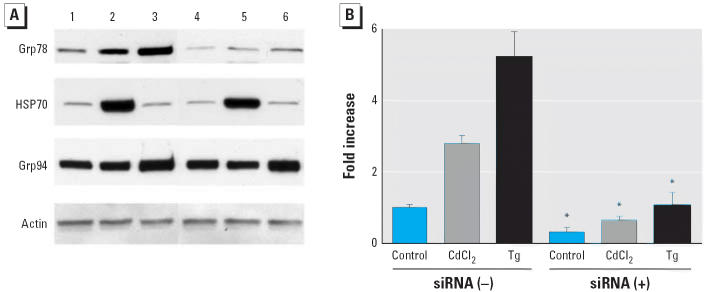
siRNA-mediated knockdown of Grp78. (*A*) Western immunoblotting of cell lysates from LLC-PK1 cells transfected without (lanes 1–3) or with (lanes 4–6) siRNA against the *Grp78* gene and incubated with 10μM CdCl_2_ (lanes 2 and 5) or 1 μM thapsigargin (Tg) (lanes 3 and 6) for 12 hr. Results shown are representative immunoblots obtained with Grp78, HSP70, Grp94, and actin antibodies. (*B*) Densitometric analysis of Grp78 protein from the immunoblot shown in (*A*). The control value (untreated cells without siRNA transfection) was set to 1; values are mean + SD of three experiments. *p < 0.001 compared with corresponding treatment without siRNA transfection.

**Figure 7:**
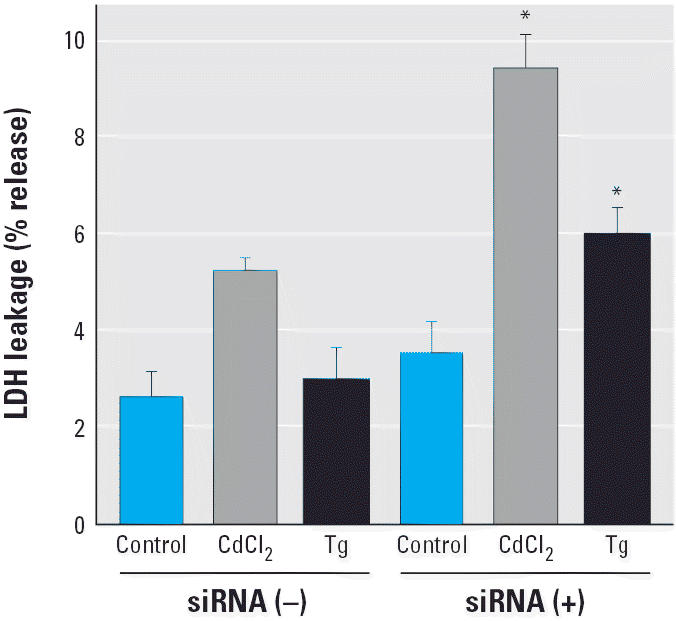
Effects of Grp78 knockdown on the cytotoxicity of CdCl_2_. LDH leakage was determined in LLC-PK1 cells transfected without or with siRNA against the *Grp78* gene and incubated with 10 μM CdCl_2_ or 1 μM thapsigargin (Tg) for 12 hr. Values are mean + SD of four determinations; results shown are representative of three experiments. *p < 0.001 compared with corresponding treatment without siRNA transfection.

To determine cellular damage, LLC-PK1 cells were incubated with CdCl_2_ or thapsigargin, an ER stressor that specifically inhibits ER Ca^2+^-ATPase (Thastrup et al. 1990), for a longer time (12 hr). Treatment with thapsigargin increased the level of Grp78 protein more markedly than did CdCl_2_ treatment (Figure 6A,B). Transfection with siRNA targeted against the porcine *Grp78* gene suppressed the levels of Grp78 protein in control, CdCl_2_-treated, and thapsigargin-treated cells by 67, 77, and 80%, respectively (Figure 6A,B). In contrast, no significant changes were found in the levels of HSP70 and Grp94 proteins by siRNA transfection, whereas treatment with CdCl_2_ and thapsigargin induced the expression of HSP70 and Grp94, respectively (Figure 6A). The knockdown of *Grp78* expression increased LDH leakage caused by treatment with CdCl_2_ and thapsigargin by 1.8- and 2.0-fold, respectively (Figure 7). After incubation with 10 or 20 μM CdCl_2_ for 12 hr, the cell viability assayed with trypan blue exclusion was 73.8 ± 8.6% at 10 μM without siRNA transfection, 60.1 ± 9.3% at 10 μM with transfection, 26.1 ± 5.7% at 20 μM without transfection, and 22.2 ± 4.0% at 20 μM with transfection (mean ± SD of five experiments).

## Discussion

The present study showed that treatment with CdCl_2_ induced the accumulation of ER chaperone protein Grp78 in a time- and dose-dependent manner in LLC-PK1 cells. The level of Grp78 mRNA was also elevated in response to CdCl_2_ exposure. Thus, cadmium exposure could cause the induction of *Grp78* gene expression, a marker for the ER stress response (UPR) (Lee 2001), in this renal epithelial cell. In addition to cadmium, treatment with thiols, iodoacetamide, *tert*-butylhydroperoxide, and sulfamethoxazole hydroxylamine has been reported to induce *Grp78* expression in LLC-PK1 cells (Halleck et al. 1997; Liu et al. 1997; Ryan et al. 2005), suggesting that ER might be an intracellular sensor of various nephrotoxic chemicals. Cadmium produces reactive oxygen species such as superoxide radical, hydroxyl radical, and nitric oxide (Valko et al. 2005) and reacts with nucleophilic ligands of target molecules (Goering et al. 1995). Cadmium toxicity in yeast has been reported to be mediated through the formation of abnormal proteins that were eliminated by the ubiquitin system (Jungmann et al. 1993). Cadmium has also been reported to mobilize Ca^2+^ from intracellular stores (Benters et al. 1997; Smith et al. 1989). Taken together, the accumulation of abnormally folded protein and the depletion of Ca^2+^ stores in the ER might underlie the mechanisms of *Grp78* expression in LLC-PK1 cells exposed to CdCl_2_. Because the level of Grp94, the most abundant glycoprotein in the ER (Lee 2001), was not changed clearly by CdCl_2_ exposure, the expression of *Grp78* and *Grp94* might be regulated by distinct mechanisms.

The transcriptional activation of the *Grp78* promoter by ER stress depends on site-1 protease– and site-2 protease–mediated proteolytic cleavage of the transcriptional factor ATF6, which specifically targets the ER stress elements (Lee et al. 2002; Ye et al. 2000). On the other hand, another transcriptional factor, ATF4 (also known as CREB2), can bind to an ATF/CRE-like site upstream of the ER stress elements in the mammalian *Grp78* promoter (Luo et al. 2003). In the present study, although the levels of the cleaved and the uncleaved forms of ATF6 were not changed (data not shown), ATF4 protein levels increased dramatically with increased time of exposure to CdCl_2_, as has been reported in mouse Hepa cells (He et al. 2001). These findings suggest that the ATF4 pathway might play a role in CdCl_2_-induced transcriptional activation of the *Grp78* gene at least in LLC-PK1 cells.

Upon ER stress, the double-stranded RNA-activated protein kinase–like ER kinase (PERK) (also known as pancreatic eIF2α kinase or PEK), an ER-resident trans-membrane protein, oligomerizes and phosphorylates eIF2α at serine 51 (Schröder and Kaufman 2005). The phosphorylation of eIF2α leads to inhibition of translation initiation by preventing the association of mRNA with ribosomal 60S and 40S subunits (Mori 2000). In contrast to most proteins, ATF4 circumvents this translation block because it has upstream open reading frames in its 5′ untranslated region that are bypassed only when eIF2α is phosphorylated (Harding et al. 2000; Rutkowski and Kaufman 2004). In LLC-PK1 cells exposed to CdCl_2_, the phosphorylation of eIF2α protein on serine 51 was found in advance of the accumulation of ATF4 protein. Although the level of ATF4 mRNA increased mildly in response to CdCl_2_ exposure, the inhibition of transcription by actinomycin D failed to suppress CdCl_2_-induced ATF4 expression, suggesting that posttranscriptional and, in part, transcriptional mechanisms are involved. In contrast, the inhibition of protein synthesis by cyclo-heximide suppressed ATF4 expression completely. Therefore, cadmium might induce the expression of *Grp78* via phosphorylation of eIF2α and resultant translation of ATF4 in LLC-PK1 cells. In addition to PERK, the heme-regulated inhibitor (HRI), the double-stranded RNA-activated protein kinase, and the general control of amino acid biosynthesis kinase are known to phosphorylate eIF2α in mammalian cells (Kaufman 1999; Rutkowski and Kaufman 2004). After exposure to CdCl_2_, an HRI-related kinase, Hri2p, induced the phosphorylation of eIF2α in yeast (Zhan et al. 2004). It remains to be determined which kinase(s) is responsible for cadmium-induced eIF2α phosphorylation in LLC-PK1 cells.

When the effects of heavy metals (10 μM) on the levels of Grp78, ATF4, and phospho-eIF2α proteins were compared in LLC-PK1 cells, another nephrotoxic heavy-metal compound, HgCl_2_, also could increase the levels of these proteins, but less significantly than did CdCl_2_. In contrast, the viability of cells treated with HgCl_2_ was more severely reduced than with CdCl_2_ (*p* < 0.05). In cells treated with 10 μM MnCl_2_, ZnCl_2_, or PbCl_2_ or 1 μM HgCl_2_ (data not shown), no significant alteration of proteins expression or cellular damage was observed. These findings suggest that heavy-metal–induced expression of Grp78 protein and its upstream regulators were not caused merely by cellular damage. The different expression of Grp78 in LLC-PK1 cells after treatment with heavy metals might be related to their distinct intracellular accumulation and biochemical properties.

To clarify the biologic significance of cadmium-induced Grp78 expression, LLC-PK1 cells were transfected with siRNA against the porcine *Grp78* gene and then exposed to CdCl_2_ or thapsigargin, an inducer of Grp78 without affecting HSP70 synthesis (Elia et al. 1996). Compared with cells without siRNA transfection, the cellular damage induced by either CdCl_2_ or thapsigargin was more severe in Grp78 knockdown cells. Although the effects of silencing of *Grp78* expression on cadmium cytotoxicity were relatively small (1.8-fold increase in LDH leakage and 18.6% reduction in the trypan blue exclusion assay) in the present study, it has also been reported that LLC-PK1 cells expressing an antisense *Grp78* construct were more susceptible to the cellular damage induced by hydrogen peroxide (Hung et al. 2003), iodoacetamide (Liu et al. 1997), and *tert*-butylhydroperoxide (Liu et al. 1998). These data and our results indicate that the expression of Grp78 plays a role in the protection against nephrotoxic insults including cadmium exposure, at least partially, in LLC-PK1 cells. In addition to its functions as ER molecular chaperone and Ca^2+^-binding protein (Lee 2001), Grp78 has been suggested to suppress oxyradical accumulation and mitochondrial dysfunction (Yu et al. 1999), repair DNA damage (Zhai et al. 2005), and inhibit caspase-7 and caspase-12 activation (Rao et al. 2002; Reddy et al. 2003). Additional studies, including animal models, are required to further reveal the protective role of cadmium-induced *Grp78* expression in the proximal tubular cells.
